# The potential of future light sources to explore the structure and function of matter

**DOI:** 10.1107/S2052252514024269

**Published:** 2015-02-03

**Authors:** Edgar Weckert

**Affiliations:** a DESY, Notkestrasse 85, 22607 Hamburg, Germany

**Keywords:** synchrotron radiation, future light sources, FELs, coherent radiation

## Abstract

Structural studies in general, and crystallography in particular, have benefited and still do benefit dramatically from the use of synchrotron radiation. Low-emittance storage rings of the third generation provide focused beams down to the micrometre range that are sufficiently intense for the investigation of weakly scattering crystals down to the size of several micrometres. Even though the coherent fraction of these sources is below 1%, a number of new imaging techniques have been developed to exploit the partially coherent radiation. However, many techniques in nanoscience are limited by this rather small coherent fraction. On the one hand, this restriction limits the ability to study the structure and dynamics of non-crystalline materials by methods that depend on the coherence properties of the beam, like coherent diffractive imaging and X-ray correlation spectroscopy. On the other hand, the flux in an ultra-small diffraction-limited focus is limited as well for the same reason. Meanwhile, new storage rings with more advanced lattice designs are under construction or under consideration, which will have significantly smaller emittances. These sources are targeted towards the diffraction limit in the X-ray regime and will provide roughly one to two orders of magnitude higher spectral brightness and coherence. They will be especially suited to experiments exploiting the coherence properties of the beams and to ultra-small focal spot sizes in the regime of several nanometres. Although the length of individual X-ray pulses at a storage-ring source is of the order of 100 ps, which is sufficiently short to track structural changes of larger groups, faster processes as they occur during vision or photosynthesis, for example, are not accessible in all details under these conditions. Linear accelerator (linac) driven free-electron laser (FEL) sources with extremely short and intense pulses of very high coherence circumvent some of the limitations of present-day storage-ring sources. It has been demonstrated that their individual pulses are short enough to outrun radiation damage for single-pulse exposures. These ultra-short pulses also enable time-resolved studies 1000 times faster than at standard storage-ring sources. Developments are ongoing at various places for a totally new type of X-ray source combining a linac with a storage ring. These energy-recovery linacs promise to provide pulses almost as short as a FEL, with brilliances and multi-user capabilities comparable with a diffraction-limited storage ring. Altogether, these new X-ray source developments will provide smaller and more intense X-ray beams with a considerably higher coherent fraction, enabling a broad spectrum of new techniques for studying the structure of crystalline and non-crystalline states of matter at atomic length scales. In addition, the short X-ray pulses of FELs will enable the study of fast atomic dynamics and non-equilibrium states of matter.

## Abstract 2

The new generation of synchrotron radiation light sources will drive analytical methods for the determination of the structure and function of molecules and crystals towards new limits in sample size and temporal resolution.

## 1. Introduction   

Advances in many fields of science, which are the foundation of new technologies, depend critically on a detailed understanding of the properties and function of molecules, crystals and matter in general. Independent of whether the research is driven by curiosity or by specific scientific or technological questions, the basis for such an understanding requires, in most cases, knowledge of the atomic and electronic structure and of its dynamics on all relevant length and time scales. During the last two decades, synchrotron radiation (SR) based techniques have evolved into indispensable analytical tools to provide this information. In particular, the advent of SR sources of the so-called third generation boosted this development dramatically. A few examples: (i) about 90% (http://biosync.sbkb.org) of all new structures in macromolecular crystallography (MX) deposited in the Protein Data Bank (PDB; Bernstein *et al.*, 1977 ▶) are based on SR data collections; (ii) other techniques like catalysis research based on absorption spectroscopy experiments depend almost entirely on the use of SR; (iii) the same holds for biological small-angle X-ray scattering (bioSAXS) or coherent diffractive imaging (CDI) experiments; and (iv) in MX, experimental techniques and data evaluation software at modern third-generation SR sources have reached an amazingly high degree of maturity and have led to the solution of some very challenging scientific questions (Deisenhofer *et al.*, 1985 ▶; Schluenzen *et al.*, 2000 ▶; Cherezov *et al.*, 2007 ▶).

Currently operating SR sources still have intrinsic limitations in one or another beam parameter that can limit some of the experiments. New schemes are discussed in the community and upgrade plans have been discussed at several SR facilities for a few years or are already in a concrete planning and approval stage. In addition, totally new concepts like energy-recovery linac sources (ERLs) are being discussed at various places and X-ray free-electron lasers (XFELs) have started operation recently or are under construction at several places.

In this contribution, we will discuss how the limitations of present-day SR sources affect our ability to unravel the structure and dynamics of matter, the various schemes for significant improvements and what impact future sources might have. Of all the possible techniques, we will limit ourselves to those that are relevant for structural studies.

## 2. Present SR sources and their limitations   

It is beyond the scope of this contribution to elaborate in detail all the parameters that influence the properties of SR emitted from a particle accelerator or storage ring. In the following, we will concentrate on those parameters that are directly relevant for experimentalists. A concise description is given by Thompson *et al.* (2009 ▶), on which all the following equations concerning the radiation properties of undulators will be based.

### 2.1. Basics of the generation of SR   

The critical energy *E*_c_, which is the energy within the spectrum of an SR source where the integrated spectral power below and above *E*_c_ is the same, depends on the particle energy *E* and the deflecting magnetic field strength *B* as
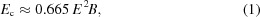
where *E*_c_ is in units of keV, *E* in GeV and B in T. Thus, for ångström-wavelength X-ray photons of several keV energy, a particle energy of several GeV is needed for the usual magnetic field strengths of the order of 1 T that can be obtained easily by permanent or electromagnets. The lower the particle energy the higher is the required magnetic field, which leads to the application of superconducting magnets up to about 7 T field strength in low-energy storage rings. The opening angle of the radiation cone of a single particle travelling at an energy *E* is given by 1/γ using
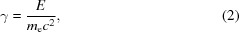
with *m*_e_ the rest mass of an electron and *c* the speed of light.

Besides the photon energy, the number of photons per unit time on the sample is probably the second most important quantity an experimentalist is interested in. Let us assume that the samples of interest are small, *e.g.* in the range of several micrometres to the sub-millimetre range. Under these conditions the relevant performance quantity is the number of photons per phase space volume, which is generally called spectral brightness. It measures the number of photons per unit time, per photon source area, per source solid angle and for a certain bandwidth (BW), given in the units photons (mm^2^ mrad^2^ s 0.1%BW)^−1^. At present, the highest spectral brightness is achieved by special magnetically periodic insertion devices called undulators (Motz, 1951 ▶; Clark, 2004 ▶). In these devices the particle beams are forced on a slalom course by a sequence of differently poled magnets. An important parameter in this context is the deflection parameter
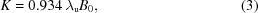
with λ_u_ the magnetic period of the undulator in cm and *B*_0_ the maximum magnetic field amplitude in T. The maximum deflection of the electron beam inside an insertion device is about *K*/γ. An undulator is characterized by *K* ≃ 1, which will result in constructive interference of the radiation from an individual electron emitted at the different poles of an undulator, leading to strong maxima in the radiated photon spectrum (harmonics) for those photon wavelengths λ_*n*_ fulfilling the resonance condition (for the zero-offset direction)
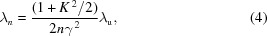
with *n* the order of the harmonics. Insertion devices with *K* >> 1 are called wigglers and will not be discussed here.

The flux density of an undulator in practical units is given by
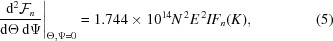
with Θ and Ψ the horizontal and vertical take-off angles, respectively, *N* the number of undulator magnetic poles, the current *I* in the storage ring in amps, *E* in GeV and *F*_*n*_(*K*) a dimensionless function with |*F*_*n*_(*K*)| ≲ 0.45, depending on the values of *K* and *n*. In general, only odd harmonics are observable on the axis of an undulator. A single electron of an undulator radiates strongly in a narrow cone with an opening angle of
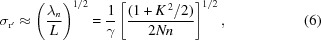
with *L* = λ_u_ *N* the total length of the undulator. The integrated intensity over the central cone is given approximately by
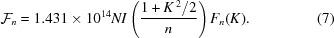
Using equations (6) and (7), equation (5) can be rewritten as
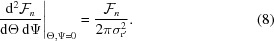



All considerations above assume that the particle beam in the storage ring is parallel, with no transverse extension and with zero energy spread. Unfortunately, real storage rings deviate from this ideal situation. They are characterized by a characteristic emittance
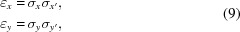
with σ_*x*,*y*_ and σ_*x*′,*y*′_ the r.m.s. values in the horizontal *x* and vertical *y* directions for the particle beam size and divergence, respectively. At all operational SR storage rings, the emittance in the horizontal direction, ∊_*x*_, is significantly larger than ∊_*y*_, with
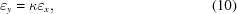
where the so-called coupling constant κ is roughly in the range 0.1–3%. This leads to considerably different source properties in the vertical direction compared with the horizontal. While the horizontal emittance ∊_*x*_ of a storage ring is fixed, machine physicists have some freedom to tailor the beam size and divergence of the particle beam by the so-called β function given by
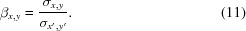
However, since the emittance is a constant, one can either make the beam larger and more parallel (high β) or smaller and more divergent (low β).

The quantity relevant for most experiments and generally used for source comparison is the average spectral brightness given by
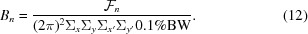
The values for the spectral brightness *B*_*n*_ refer to a 0.1% spectral bandwidth (BW) interval, which is roughly 7–10 times larger than the bandwidth after a standard Si(111) crystal monochromator. To a first approximation, the effective photon source sizes Σ_*x*,*y*_ and divergences Σ_*x*′,*y*′_, where 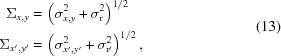
are a convolution of the sizes σ_*x*,*y*_ and divergences σ_*x*′,*y*′_ of the particle beam with the intrinsic single-electron radiation size σ_r_ and divergence σ_r′_ from an axially extended source of length *L* for a given photon wavelength λ, given by σ_r_ = (λ*L*)^1/2^/4π and σ_r′_ = (λ/*L*)^1/2^. For time-resolved experiments, it is more appropriate to consider the spectral brightness during the duration of a photon pulse. This so-called peak spectral brightness can be derived from equation (12) according to
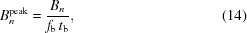
where *f*_b_ and *t*_b_ are the pulse frequency and duration, respectively.

In Table 1 ▶ the emittance values for a number of storage rings are given. All values at present-day sources are of the order of several nm rad. This means that, for a photon wavelength of about 1 Å which is characteristic for structural studies at atomic resolution, the spectral brightness according to equation (12) is mainly dominated by the emittance of the storage rings, since the corresponding σ_*x*,*y*,*x*′,*y*′_ values, especially in the horizontal direction, are considerably higher than the corresponding σ_r,r′_ values. Table 1 ▶ also gives the wavelength, according to
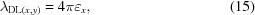
at which the spectral brightness starts to become dominated by the characteristic single-electron properties. We call this the diffraction limit, which means that, for all wavelengths λ ≥ λ_DL(*x*,*y*)_, the source can be considered to radiate almost coherently. In addition, reducing the emittance significantly below this value will not lead to any further increase in the spectral brightness.

The horizontal emittance ∊_*x*_ is a characteristic equilibrium quantity for the stored beam of each storage ring. The typical time until the injected charge is damped to this value by emission of SR is of the order of several milliseconds. For each storage ring, the emittance can be calculated precisely, given by
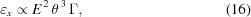
[see *e.g.* Wiedemann (2007 ▶), Bilderback *et al.* (2005 ▶) and Borland (2013 ▶)]. Here, Γ denotes a quantity depending on the design of the magnetic lattice of the storage ring. The angle θ denotes the angular deflection of the particle beam by an individual bending magnet.

From equations (1) and (16) it is obvious that we encounter a conflict of aims. On the one hand we need higher particle energies *E* in order to obtain X-rays in a wavelength range that allows atomic resolution structural studies according to equation (1), but this will result in a larger emittance of the storage ring according to equation (16) and, therefore, in a larger beam and a reduction in the achievable spectral brightness. On the other hand, it is also not possible to increase the magnetic field arbitrarily to produce harder X-rays, since we need to keep *K* in equation (3) roughly below 2–3 for an undulator to work properly in order to provide a high spectral brightness, since for technical reasons λ_u_ cannot be small and *B*_0_ very large at the same time. This technological challenge has been partly addressed by the use of *in vacuo* and cryogenic *in vacuo* undulators (Hara *et al.*, 1998 ▶, 2004 ▶; Chavanne *et al.*, 2008 ▶) for which, due to a smaller possible magnetic gap, slightly higher magnetic fields *B*_0_ can be achieved with correspondingly shorter magnetic periods λ_u_. Ongoing developments to use superconducting undulators (Rossmanith *et al.*, 2002 ▶; Ivanyushenkov & Harkay, 2014 ▶) will certainly push the limits for smaller magnetic periods and higher fields at the same time. Since *E*_c_ in equation (1) depends only linearly on *B* and quadratically on *E*, increasing the particle energy is significantly more efficient for the generation of hard X-rays than increasing the magnetic field. However, from equation (16) it is also obvious that, in principle, it is possible to obtain a higher energy storage ring with a small emittance by keeping θ small, which means one needs to use a large number of bending magnets with a small deflection angle, which will be discussed in the next section. At current higher energy sources with two bending magnets per cell, this has been addressed by increasing the number of cells and thus reducing the deflection angle per bending magnet, *e.g.* ESRF (32 cells), APS (40 cells) and SPring-8 (48 cells).

The radiation generated by individual electrons in an undulator is uncorrelated. In this sense an SR source is ideally incoherent. However, due to the extreme collimation of the radiation cone with an opening angle of the order of 1/γ, the light arriving at the sample is partially coherent, due to the large source-to-sample distance and the small source size. The coherent flux of an undulator is given by
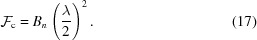
For the best SR sources operating at present, 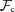 is still below 1% of the total flux provided (Vartanyants & Singer, 2010 ▶).

The energy width of an undulator harmonics line is given by
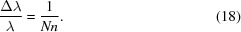
Usually, Δλ/λ ≃ 1%. Equation (18) indicates that, with increasing *N*, a more monochromatic undulator harmonics would be obtained. However, since for the particle beam of a storage beam Δ*E*/*E* ≃ 0.001, the delivered relative spectral width is a convolution of the energy resolution due to equation (18) with the energy spread of the particles. If the particle energy spread dominates, the relative spectral width will be roughly twice the Δ*E*/*E* of the particle beam, due to equation (4). For a given Δλ/λ, the longitudinal coherence length *l*_c_ is given by
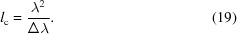
This means that *l*_c_ for 1 Å radiation after an Si(111) monochromator is only roughly 1 µm. This will be of importance for coherence experiments at higher *q* ranges, where the path differences of interfering beams will have to be taken into account.

The achievable temporal resolution at a storage-ring source is limited by the length of the electron bunches, which is determined by the properties of the RF system that is needed to restore the energy the particles have lost due to emitting SR. The r.m.s. values for the bunch length are, in general, of the order of 20–50 ps, thus limiting the temporal resolution to these values. The single-bunch intensities at a storage ring can be up to 10^9^ photons if the complete spectrum of an undulator harmonics can be transported to the sample (Cammarata *et al.*, 2008 ▶).

For lower energy storage rings, operation modes have been established for shorter pulses. In the so-called low-α mode of operation (Wiedemann, 2007 ▶; Robin *et al.*, 1993 ▶; Hama *et al.*, 1993 ▶; Abo-Bakr *et al.*, 2003 ▶), single-digit picosecond bunch length values can be obtained at beam currents of the order of 1% of the full current and a corresponding lower flux density or spectral brightness. Even shorter pulses of the order of 100 fs can be achieved by laser slicing techniques, where a short slice of an electron bunch is prepared such that the radiation of the short slice can be spatially separated from that emitted from the rest of the bunch (Zholents & Zolotorev, 1996 ▶). In this case, the achievable intensities are roughly determined by the ratio of the slice length to the total bunch length.

### 2.2. Limitations of present-day storage-ring sources   

In order to clarify the limitations we experience at present-day sources, it is probably best to consider first those cases where no limitations exist and superb experimental conditions are already provided. For all cases where the energy resolution of the incoming beam is sufficient after an Si(111) or multilayer monochromator, and for a focal spot size well above a diffraction-limited focus (Schroer & Falkenberg, 2014 ▶), today’s third-generation SR sources provide adequate spectral brightness and flux densities in their main energy range. This addresses most diffraction and absorption spectroscopy experiments in a photon energy range of, say, 5–25 keV. Just to give an example: almost no frozen protein crystal can withstand the beam of a state-of-the-art microfocus beamline for more than a few seconds at any of the newer third-generation sources without suffering severe radiation damage (Garman, 2014 ▶). However, experimental boundary conditions, like the minimum distance needed between the last focusing element and the sample due to the requirement of suitable sample environments, might limit the demagnification ratio and, in turn, the portion of the total beam cross section that can be focused for focal spot sizes in the 1 µm range. This has led to the construction of very long beamlines at present sources, like in the upgrade phase I of the ESRF (Grenoble, France). The same holds if experiments need high *q* resolution, thus requiring a small divergence of the incident beam. Another approach for increasing the working distance between a very small focus and the last optical element at the expense of a somewhat larger divergence is a double focusing scheme, achieving focal-spot sizes in the range 30–50 nm at a working distance of 0.35 m (Mimura *et al.*, 2014 ▶).

If higher flux densities at the same focal-spot sizes are required according to equation (5), this can – at least in principle – be achieved by increasing the beam current *I* or the number *N* of the magnetic poles of an undulator. Unfortunately, the latter option is restricted due to the limited monochromaticity of the particles in a storage ring, which is of the order of Δ*E*/*E* ≃ 0.001. Therefore, the *N* ^2^ increase in flux density is only valid for a limited undulator length until the increase with *N* becomes linear. As an example, for PETRA III at a 1 nm rad emittance and Δ*E*/*E* ≃ 0.0012 at about λ_u_ ≃ 30 mm, the *N* ^2^ increase in spectral brightness only holds up to an undulator length of 4 m. Due to the parabolic shape of the β function [equation (11)] in the straight sections of a storage ring, longer undulators require larger magnetic gaps and therefore larger values of λ_u_ to obtain the same *K*_max_ [equation (3)]. This limits the possibility of increased flux density, unless the electron beam is focused between different undulator segments.

The smallest possible (diffraction-limited) spot size can only be achieved if the coherent part of the radiation is accepted by the focusing element (Singer & Vartanyants, 2014 ▶; Schroer & Falkenberg, 2014 ▶). Therefore, the intensity at such a focus is at present limited to significantly less than 1% of the total intensity, which limits all experiments that need a nanofocus, such as X-ray nanoprobes or X-ray scanning microscopes. The limited coherent flux also severely limits a number of other experimental techniques, such as X-ray correlation spectroscopy (XPCS; Grübel & Zontone, 2004 ▶; Grübel *et al.*, 2008 ▶) and coherent diffractive imaging (CDI) and tomography (Miao, Kirz & Sayre, 2000 ▶; Williams *et al.*, 2003 ▶; van der Veen & Pfeiffer, 2004 ▶; Schroer *et al.*, 2008 ▶). This holds even more for those cases where not only the static structure but also its (slow) dynamics need to be studied. For XPCS at higher *q* values, monochromatic radiation is needed such that the differences in path length for different spots at the sample to the detector are smaller than the longitudinal coherence length [equation (19)]. This criterion additionally reduces the available flux for such experiments. Further on, coherent X-rays are starting to open up totally new ways for the analysis of disordered materials by angular X-ray cross-correlation methods, providing information on the local order of dis­ordered materials (Wochner *et al.*, 2009 ▶; Altarelli *et al.*, 2010 ▶; Kurta *et al.*, 2012 ▶; Kurta, Dronyak *et al.*, 2013 ▶; Kurta, Ostrovskii *et al.*, 2013 ▶). This method is also mainly limited by the available coherent flux.

As mentioned above, the spectral width of an undulator harmonics line is of the order of 1%. Most experiments at SR sources use a Si(111) monochromator with an energy resolution of roughly 10^−4^, so 99% of the available intensity of an undulator harmonics line cannot be used. The situation is even worse for all those experimental techniques that require a very well defined incoming photon beam energy, such as high-resolution diffraction and absorption spectroscopy experiments. Even more severely affected are all the inelastic X-ray and nuclear scattering techniques, which need better monochromaticity by at least another factor of ∼100.

Dynamic studies at SR sources are possible in principle if the single-bunch intensity is sufficient to allow for experiments that can be repeated multiple times, and if the required temporal resolution can be met by the intrinsic bunch length at a synchrotron storage ring. All the special techniques mentioned above, like low-α mode or femto-slicing, can certainly serve some applications but are in general limited by the achievable single-bunch intensity.

## 3. Next-generation sources   

In this section, both recent developments and new ideas for SR sources with dramatically improved source parameters will briefly be discussed, as well as new results and their expected impact on various scientific fields.

### 3.1. Towards diffraction-limited light sources   

As described in §2, the spectral brightness of an SR source grows with a smaller emittance of the storage ring towards the values given by the diffraction limit. If we further assume that the particle energy is more or less fixed by the main applications the source is targeted for by equation (1), then from equation (16) a means of reducing emittance is to decrease the angle θ for the deflection of the particle beam by individual bending magnets. This can be achieved in two ways.

#### 3.1.1. Damping wigglers   

The straightforward method is to use a traditional lattice design with two bending magnets per cell (double-bend achromat, DBA) and to install a number of damping wigglers into some of the straight sections (Wiedemann, 1988 ▶). Examples of this approach are the PETRA III SR source at DESY in Hamburg, Germany (Weckert *et al.*, 2004 ▶), and the new NSLS-II SR source at BNL, USA (NSLS-II, 2010 ▶). By this approach the emittance can practically be lowered by roughly a factor of 4–5 compared with the corresponding DBA lattice. The performance values of these two examples are shown in the middle of Table 1 ▶. The advantages of this approach are that, in quite a straightforward manner, the emittance can be reduced by a significant amount and the damping wigglers can be used as sources for applications requiring extremely high photon energies. The drawbacks of this approach are the higher power consumption, due to radiation loss in the damping wiggler sections, and that space otherwise available for undulator insertion devices will be taken by damping wigglers if their radiation properties are not useful for the targeted applications.

#### 3.1.2. Multibend achromat storage rings   

Another method for lowering the emittance is to split the two bending magnets normally used per cell at present SR sources into a larger number of magnets. Since the deflection angle θ per bending magnet contributes with the third power to the emittance, according to equation (16), a considerable reduction in emittance can be achieved. Even though there are a number of technical challenges that still need to be overcome, this scheme provides the means for pushing the diffraction limit to higher photon energies and possibly into the harder X-ray regime. The first project that is being built according to this scheme is the MAX IV storage ring at MAX-lab in Lund, Sweden (MAX IV, 2014 ▶), employing a seven-bend achromat lattice which will result in an emittance of 0.2–0.3 nm rad. All further parameters can be found in Table 1 ▶. As soon as MAX IV starts operating it will be the synchrotron storage ring with the smallest emittance, at least for this decade.

The push towards lower emittance has picked up huge momentum during the last few years. A dedicated series of workshops has been organized to discuss the science case, storage-ring lattice design and X-ray optics. All of the currently operating large-scale high-energy facilities, ESRF (France; Farvacque *et al.*, 2013 ▶; Revol *et al.*, 2013 ▶), APS (USA; Borland, 2014 ▶) and SPring-8 (Japan), are working out concrete upgrade plans. Proposals are also worked out for the refurbishment of existing large storage rings, like PEP-X at SLAC (Stanford, USA) and PETRA III. For the 3 GeV class of storage rings, concrete projects have also already been set up, like SIRIUS (Campinas, Brazil; LNLS, 2014 ▶), or design studies are under way. In the lower part of Table 1 ▶, the expected performance parameters of some of these new multibend achromat sources are listed, as far as they are available. The most advanced in this process is the ESRF upgrade phase II project, which received unanimous support for its implementation by the ESRF council in June 2014. Provided everything works out as planned, the upgraded ESRF will start user operation in 2020.

#### 3.1.3. Science drivers for low-emittance SR storage rings   

In terms of total flux density according to equation (5), the planned upgrades will not be significantly different from the best present-day sources. Even the expected heat load of an unfocused beam on the X-ray optical systems will be comparable with what can be handled now. The main contribution to the increase in spectral brightness will be due to the significantly smaller source sizes [equations (12) and (13)]. From Table 1 ▶ it becomes obvious that all the new diffraction-limited SR (DLSR) source projects will provide considerably higher spectral brightness and coherent flux compared with a typical third-generation source at present, with an expected increase by roughly a factor of 30–100. The values for λ_Dl*x*_ also indicate that, for the horizontal direction, all new designs will make a huge step towards the diffraction limit into the harder X-ray regime, although none of them will really get close to something like 1 Å. Depending on the planned vertical to horizontal coupling ratio [equation (10)], all presently operating third-generation sources are already close to the diffraction limit in the vertical direction for wavelengths in the 1 Å regime, and it is expected that all new multibend achromat designs will operate at κ values [equation (10)] which provide conditions for fully coherent radiation at 1 Å or even shorter in the vertical direction.

*Experiments requiring a focus larger than the diffraction limit.* For this class of experiments, the smaller source size of the planned upgraded sources will provide some benefits. For the same focal spot size, the incoming beams are more parallel and the working distance between the last focusing X-ray optical element and the sample will be larger. This is especially interesting for experiments that need bulky sample environments, *e.g.* in high-pressure research using diamond anvil cells. The increased spectral brightness will translate directly into a corresponding increase in focal flux density, allowing for significantly faster experiments, which will be especially important for studies of slow (compared with the bunch length of roughly 100 ps) dynamics and kinetic effects or studies of minute changes of signals that are simply not currently accessible due to a lack of counting statistics. For crystallographic studies in the field of macromolecular crystallography, experiments derived from techniques developed for X-ray free-electron lasers (XFELs) like serial crystallography on microcrystals (Gati *et al.*, 2014 ▶) will also profit greatly from these improved experimental conditions.

*Experiments needing a fully coherent beam.* The expected increase in spectral brightness by a factor of up to 100 will translate directly to the same increase in the available coherent flux in a diffraction-limited focus. All methods mentioned above, such as CDI, will benefit directly from this development. In particular, X-ray ptychography (Rodenburg *et al.*, 2007 ▶; Schropp *et al.*, 2012 ▶, 2013 ▶) and X-ray ptychography tomography (Dierolf *et al.*, 2010 ▶; Diaz *et al.*, 2012 ▶; Trtik *et al.*, 2013 ▶; Holler *et al.*, 2014 ▶), which require scanning of the sample in steps of a fraction of the diameter of the coherent focus and a large number of projections, are extremely time consuming these days and would greatly benefit from the dramatically increased coherent flux, bringing down the time for such experiments from hours at present to minutes, or allowing for increased resolution, which is mainly limited by counting statistics at present. Such a development would bring us much closer to a routinely applicable X-ray three-dimensional microscope; it would never be able to compete with electron microscopy in terms of resolution but it would allow the study of thicker samples under relevant application conditions without special preparation at a resolution of the order of 5 nm or better.

*Experiments exploiting partially coherent beams.* Most X-ray ‘coherence’ experiments at present-day storage-ring sources apply a partially coherent beam. Due to the low coherent flux, experimentalists always have to trade the useable flux, requiring a larger aperture, and the interference or speckle contrast, needing a smaller aperture, for selection of the coherent part of the beam. Those experiments that, for reasons of counting statistics or sample volume averaging, need to sample volumes larger than the fully coherent part of the beam will benefit in two ways from the beam properties of the new storage ring. Assuming a fixed probe volume, the intensity in that volume will be higher due to the higher spectral brightness and, at the same time, the interference contrast will increase due to the higher degree of coherence as a result of the smaller horizontal source size. Under this category fall XPCS and, to some extent, phase-contrast imaging experiments. For XPCS this means that significantly shorter timescales than today will be accessible.

### 3.2. Energy-recovery linac sources   

#### 3.2.1. Operation principle   

The equilibrium emittance in equation (9) of a newly injected beam in a storage ring is only achieved after the so-called damping time, which is of the order of milliseconds. This holds for an injected beam with either a larger or a smaller emittance compared with the equilibrium value. The idea of an energy-recovery linac (ERL) driven source is to inject a very low emittance beam from a linac into a ring or an arc and to extract the electron bunches after one or a few turns. With all parameters adjusted properly, the emittance of the injected beam should almost be maintained during the first turn. On extraction, the energy of the used electron bunches needs to be recovered, since otherwise, for operating conditions comparable with present sources in terms of particle energy and current, the power consumption of such a facility would be of the order of hundreds of megawatts. This energy recovery can be achieved by feeding the used beam back into the linac with a phase shift of π. Here it will be decelerated, thereby storing the beam’s energy in the cavities of the linac to be used for the acceleration of a new beam. This scheme, first proposed by Tigner (1965 ▶), in principle allows for an almost complete recovery of the beam’s energy if a high-fidelity superconducting linac is used (Gruner & Tigner, 2001 ▶). Several ERLs are already in operation with particle energies in the range of tens of MeV, driving FELs in the THz regime. Energy recovery up to a beam power of 1.3 MW has been demonstrated to date (Merminga, 2007 ▶). An overview of the most important ERL activities is given by Nakamura (2012 ▶). A comprehensive summary of the challenges of a high-energy ERL source and the corresponding science case has been compiled by the CHESS team at Cornell University (Bartnik *et al.*, 2013 ▶; Hoffstaetter *et al.*, 2013 ▶).

#### 3.2.2. Technical challenges   

For ERLs the technical challenges are similar to FELs, because the emittance of the electron bunches produced by the injector electron gun determines the properties of the particle beam. The difference is that an ERL gun has to sustain an average beam current about a factor of 1000 higher than a FEL, which poses considerable technical challenges especially for the cathode material (Siggins *et al.*, 2001 ▶; Dunham *et al.*, 2007 ▶). This is especially critical if the ERL aims to produce diffraction-limited photon beams in the X-ray regime, requiring an even lower normalized emittance [equation (20)] than a FEL, due to the absence of the self-amplified spontaneous emission (SASE)-FEL process which generates a large number of photons per mode. Another challenge is the rate of energy recovery, which directly affects the operation budget. To date, the highest power beam de- and accelerated in an ERL was about 1.3 MW at Jefferson Laboratory, USA (Merminga, 2007 ▶). A beam power at least two orders of magnitude higher is needed for an X-ray ERL. Since an ERL is driven by a linac, the electron bunches can in principle be very short, but the emission of coherent SR (CSR) at each bending magnet will limit the accessible bunch lengths to the range of about 1–2 ps (Mayes & Hoffstaetter, 2010 ▶). For significantly shorter bunch lengths, some degradation of the emittance and bunch length is expected after the first few bending magnets.

#### 3.2.3. Comparison with storage-ring and FEL sources   

The key parameters of some planned ERL projects are compared in Table 2 ▶. Compared with storage-ring sources, ERLs have an almost round beam, which means that ∊_*x*_ ≃ ∊_*y*_. The emittance in an ERL is determined by the normalized emittance,
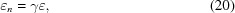
that can be achieved by the electron gun and by the maximum energy to which the electrons are accelerated in the linac. While the equilibrium emittance in a storage ring increases with *E* ^2^ [equation (16)], the geometric emittance in a linac decreases with 1/*E* according to equation (20). As is the case for FELs, the normalized emittance depends critically on the bunch charge due to space charge effects (Vashchenko, 2013 ▶). For this reason, ERLs are planned to operate in two different operation modes: (i) a low bunch charge, low-emittance and high-coherence mode, and (ii) a higher bunch charge and high-flux mode. In order to obtain a current comparable with present-day sources, bunch repetition rates of the order of 1 GHz are envisaged.

*Spectral brightness comparison.* A comparison of the parameters of projected storage rings (Table 1 ▶) with those of proposed X-ray ERLs (Table 2 ▶) reveals that the horizontal emittances are comparable, to within a factor of 2–3, for the planned high-flux ERL operation mode. The beams in an ERL will be almost round, while storage-ring beams will almost certainly have a coupling ratio [equation (10)] such that at least the vertical emittance of the present source will be achieved, *i.e.* ∊_*y*_ ≲ 35 pm rad. This means that the total four-dimensional transverse phase space of these sources would be comparable, thus providing, for the same current, similar average spectral brightness and coherent fraction values for comparable undulator parameters. Since the energy spread of an ERL is about a factor of five smaller than for a storage ring, the *N* ^2^ relation in equation (5) holds for larger *N*, enabling a more efficient increase in spectral brightness by using longer undulators and also leading to spectrally more narrow undulator lines [equation (18)]. For applications exploiting spectral brightness and coherence, all arguments made above for DLSRs (§3.1.3) also hold for ERL sources. For the high-coherence ERL operation mode, it is planned to reduce the bunch charge, and thus also the emittance, by a factor of roughly 4–10. This will also reduce the total flux by the same factor. However, since the source size is now considerably smaller, the spectral brightness and therefore the coherent flux will be comparable with the high-flux mode, albeit at a much reduced heat load on the X-ray optical elements (Bilderback *et al.*, 2010 ▶). In this operation mode, an ERL is at the diffraction limit for both transverse directions for photon wavelengths longer than 2 Å. Compared with an XFEL, the average spectral brightness of both an ERL and a DLSR is comparable with FELs with low repetition rates. The average spectral brightness of superconducting linac-driven high-repetition-rate FELs is still several orders of magnitude above these sources.

*Comparison for time-resolved studies.* For dynamic or time-resolved studies at the order of 1 ps resolution that can be carried out at GHz repetition rates, ERLs have a clear advantage compared with a DLSR, due to the significantly shorter pulse length. XPCS is certainly a technique that would benefit from such a source, as will some spectroscopic pump–probe techniques for condensed matter samples. In the latter case, the available repetition rate of the optical pump laser and the stability of the sample will limit the data-collection rate. Special ERL operation modes with even shorter pulses are also considered (Bartnik *et al.*, 2013 ▶), although these short pulses will then only be available to a limited number of beamlines before the bunch length starts to grow due to the emission of coherent SR. Due to the SASE lasing process, the peak spectral brightness of a FEL is still 4–6 orders of magnitude higher than for an ERL. This means that, for single-shot high-peak spectral brightness experiments like most of the examples cited in §3.3.2, FELs will have a distinct advantage over ERLs or DLSRs.

### 3.3. Free-electron lasers   

**Figure 1:**
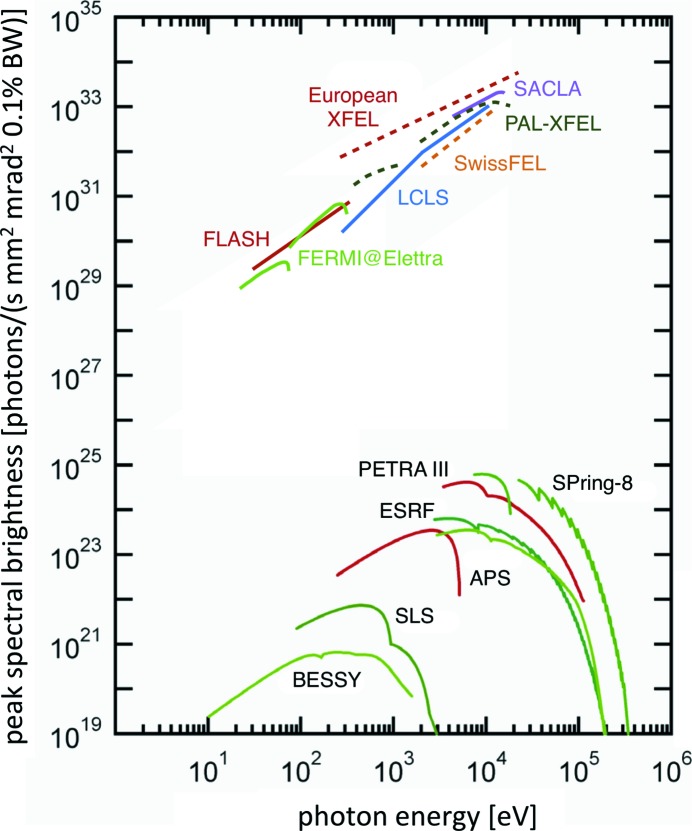
A comparison of peak spectral brightness 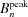 [see equation (14)] of some storage-ring and FEL sources. Reprinted with permission from Schmüser *et al.* (2014 ▶). Copyright (2014) Springer-Verlag.

During the last ten years, several free-electron lasers (FELs) (Derbenev *et al.*, 1982 ▶; Murphy & Pellegrini, 1985 ▶) in the VUV and soft X-ray regime [FLASH at DESY in Hamburg, Germany (Ackermann *et al.*, 2007 ▶), and FERMI at ELETTRA, Italy (Allaria *et al.*, 2012 ▶)] and in the harder X-ray regime [LCLS at SLAC, USA (Emma *et al.*, 2010 ▶), and SACLA at SPring-8, Japan (Ishikawa *et al.*, 2012 ▶)] have been successfully brought into operation and user experiments started. In addition, a number of FEL projects are under construction, such as the European XFEL in Hamburg, Germany (Abela *et al.*, 2007 ▶), the SwissFEL at PSI, Switzerland (Ganter, 2010 ▶), and the FEL at the Pohang Accelerator Laboratory (PAL) in Korea (Kim, Choi *et al.*, 2008 ▶). At SLAC, an extension of the present LCLS facility (LCLS II) is in preparation. At several other places new FEL facilities are in the planning stage. An overview of the technical parameters of those facilities currently operating and those under construction is given in Table 3 ▶. A comparison of the peak spectral brightness of some storage-ring and FEL sources is shown in Fig. 1 ▶.

Most FELs work in the so-called self-amplified spontaneous emission (SASE) regime (Kondratenko & Saldin, 1980 ▶; Bonifacio *et al.*, 1984 ▶; Schreiber, 2010 ▶). In order to characterize the emittance in a FEL, the normalized emittance of equation (20) is used. This quantity indicates the quality of the electron beam independent of the particle energy. In order to drive a SASE-FEL, electron bunches with ∊_*n*_ ≤ 1 mm mrad, a charge of roughly 0.02–1.0 nC, a length of 10–200 fs and an energy spread smaller than 10^−4^ are accelerated in a linac and sent through a long precisely aligned undulator. Due to the interaction of the generated spontaneous light field in the first undulator part with the particle beam within the undulator, the density of the electron bunch inside the undulator becomes modulated on the length scale of the photon wavelength [equation (4)] to which the undulator is tuned. Due to this modulation, slices of electrons in the bunch radiate almost coherently, giving rise to a dramatic increase in photon intensity (Fig. 1 ▶). The reader is reminded that the bunch lengths here are more than three orders of magnitude shorter than those discussed above for storage rings. The calculated emittances of the higher-energy FELs mentioned above at the exit of the linac are of the order of 10 pm rad in both transverse directions, providing a fully diffraction-limited beam in the 1 Å wavelength regime for the spontaneous radiation of the undulator. The coherent fraction of SASE-FEL radiation in saturation approaches 100% for a sufficiently long undulator (Saldin *et al.*, 2008*a* ▶,*b* ▶).

The small emittance and high bunch current density needed for a FEL can at present only be achieved by the use of a linac. Since the SASE process in a FEL undulator starts from noise, the output power of such a source is not constant and follows a gamma distribution (Saldin *et al.*, 1998*b* ▶,*a* ▶) depending on the number of active laser modes. For the same reason, the output radiation has a spiky structure in the frequency domain. This can be overcome by seeding the FEL. Here, the seeding wavefield has to be stronger than the spontaneous emission at the beginning of the FEL undulator. Several seeding schemes are under discussion (Reiche, 2013 ▶) and it is beyond the scope of this contribution to discuss these in detail. External seeding in the VUV range by the use of a frequency-multiplied optical laser beam has been successfully established at FERMI (Allaria *et al.*, 2012 ▶; Allaria, Castronovo *et al.*, 2013 ▶). Recently, using a high-gain harmonic generation (HGHG) scheme (Yu, 1991 ▶) in a second step, seeded radiation in the soft X-ray regime has been generated. Due to the lack of a suitable seeding source in the hard X-ray regime, self-seeding is the only option for this photon energy range. Such schemes have been implemented successfully at LCLS (Amann *et al.*, 2012 ▶) according to a proposal by Geloni *et al.* (2010 ▶).

If sufficient undulator length is available, the full potential of a FEL can be achieved by tapering the undulator such that, after saturation has been achieved, the resonance condition for the photon beam is maintained, even though energy from the particle beam has been transferred towards the photon beam (Orzechowski *et al.*, 1986 ▶). This scheme is capable of providing roughly 100 times greater spectral brightness for a spectrally narrow seeded photon beam (Serkez *et al.*, 2013 ▶; Amann *et al.*, 2012 ▶) and should also work for a pure SASE mode of operation, albeit slightly less efficiently with a gain of roughly one order of magnitude (Agapov *et al.*, 2014 ▶) compared with the non-tapered case. In addition, tapering a FEL undulator after saturation will in any case increase the coherent fraction of the SASE radiation.

#### 3.3.1. Comparison with storage-ring sources   

Compared with existing and planned storage rings, the radiation of any FEL is considerably more transversely coherent, even if it is not totally coherent in all cases (Singer *et al.*, 2012 ▶). This means that all the techniques mentioned above, like CDI and diffraction-limited focusing, benefit dramatically from these source properties and scientists have not yet really started to take full advantage of such a high degree of coherence.

The largest single pulse intensity at a storage ring is roughly 10^10^ photons for 3% spectral bandwidth (Wulff *et al.*, 2007 ▶). At XFELs, each pulse delivers 10^11^–10^13^ photons for about 0.1% spectral bandwidth. As already mentioned, the photon pulses at FELs are about 10^3^ times shorter than for a storage ring under normal operating conditions. They are also more than 10^7^ times more intense than on a slicing source. This means that scientists now have a tool to investigate matter on atomic length scales and at femtosecond time scales. This allows for the investigation not only of the static equilibrium atomic structure but also of fast dynamics and non-equilibrium states of matter. Conducting these experiments and finding appropriate triggers to start reactions (*e.g.* by a suitable femto­second optical pump laser) or to transfer matter into a defined non-equilibrium state is far from trivial at such short timescales and is part of ongoing research efforts.

The repetition rate of FELs (see Table 3 ▶) is about 20–120 Hz for linacs using normal conducting technology and up to 27 kHz for superconducting technology. Plans exist to push this up to the 1 MHz regime (Corlett *et al.*, 2012 ▶). These repetition rates have to be compared with SR storage rings, which are generally of the order of MHz. From this comparison it is immediately obvious that all FELs driven by a normal conducting linac will be used mostly, but not exclusively, for ‘low repetition’ rate and, in extreme cases, single-shot experiments. The possible higher repetition rates at superconducting linac-driven FELs are advantageous for all experiments working with extremely dilute samples or those where the sample can be exchanged sufficiently rapidly, like for a fast flowing jet.

A disadvantage of FELs is the comparatively small number of beamlines that can be operated in parallel. Switching the electron beams to more than one FEL undulator is being set up at FLASH (two FEL undulators) at DESY (Faatz *et al.*, 2010 ▶; Ayvazyan *et al.*, 2011 ▶) and the European XFEL (maximum five FEL undulators), and is planned for the upcoming LCLS II project (two FEL undulators). In all cases, the number of bunches accelerated in the linac will then be distributed to several FEL undulators, lowering the effective repetition rate at each experiment.

#### 3.3.2. Science and developments at FELs   

Since the first FEL started user operation in 2005, a number of ground-breaking experiments have been carried out successfully and an entire new field has evolved, or more precisely is still in the process of evolving. Within the framework of this contribution, it is not possible to cover properly the entire literature published in this field. Nevertheless, an attempt will be made to trace some of the most important steps and experiments of these exciting new developments.

The entire field is characterized by an ongoing development of the sources, experimental schemes, detection and data-handling issues, as well as data-analysis methods. It will still take several years until the majority of experiments at FELs are as mature as what we are used to at third-generation sources today. Since the almost coherent FEL radiation can be focused efficiently, hitherto unprecedented photon flux densities can be generated, which immediately raises questions about the stability of X-ray optical components under these conditions. These experiments started first at FLASH (Steeg *et al.*, 2004 ▶; Stojanovic *et al.*, 2006 ▶; Hau-Riege *et al.*, 2007 ▶; Chalupský *et al.*, 2009 ▶) for mirrors and mirror coatings, and for multilayer optics (Khorsand *et al.*, 2010 ▶; Sobierajski *et al.*, 2011 ▶) in the soft X-ray regime. For the harder photon energy regime, corresponding experiments were carried out at LCLS (Gaudin *et al.*, 2012 ▶; Uhlén *et al.*, 2013 ▶), and at SACLA for focused beams (Yumoto *et al.*, 2012 ▶; Koyama *et al.*, 2013 ▶; Mimura *et al.*, 2014 ▶). At SACLA, considerable work has also been carried out on the quality of X-ray mirrors (Mimura *et al.*, 2008 ▶), which is extremely important for maintaining the wavefront of the almost totally coherent FEL beam through the entire X-ray optical system towards the sample.

Experiments at FELs targeting timescales of the order of femtoseconds require the development of a number of techniques that have no analogy at SR sources. Due to the SASE process, there is some randomness for the photon pulses in terms of profile shape, length and arrival time. Most crucial for time-resolved studies are the pulse length and the arrival time jitter with respect to an optical laser. Terahertz streaking of photoelectrons generated by the FEL pulse passing through a low-pressure gas target seems to be one of the most promising methods for non-invasive measurements of these quantities (Fruehling *et al.*, 2009 ▶; Tavella *et al.*, 2011 ▶; Grguras *et al.*, 2012 ▶). Other methods under development are cross-correlation methods, which are achieved by shining a portion of the beam of the optical pump–probe laser and the FEL at an oblique angle onto the same spot of a suitable material, which changes its optical properties on interaction with the FEL pulses, thus providing relative timing information (Gahl *et al.*, 2008 ▶; Beye *et al.*, 2012 ▶; Riedel *et al.*, 2013 ▶; Harmand *et al.*, 2013 ▶). The derived timing signal can then be used for temporal sorting of the results of the individual pulses, effectively providing a timing accuracy limited by the cross-correlation timing signal.

FEL radiation shows a very high degree of coherence that needs to be quantitatively characterized for almost any experiment exploring these properties. Several methods have been successfully applied to determine the coherence properties of various FELs, such as evaluation of the interference contrast using a Young’s double-slit experiment (Singer *et al.*, 2008 ▶, 2012 ▶; Vartanyants *et al.*, 2011 ▶), exploitation of the statistical properties of light according to the method proposed by Hanbury-Brown & Twiss (1956 ▶) applied to FEL radiation (Singer *et al.*, 2013 ▶), and analysis of speckle patterns of colloidal particles (Gutt *et al.*, 2012 ▶; Lehmkühler *et al.*, 2014 ▶). For many imaging experiments, information on the entire wavefront of the incident beam is important, and this is accessible by various wavefront analysis techniques, *e.g.* by a Hartmann wavefront sensor (Bachelard *et al.*, 2011 ▶) or by interference effects due to a defined phase grating (Rutis­hauser *et al.*, 2012 ▶).

Among the new experimental developments are the first two wave-mixing experiments involving X-rays to unravel changes in the electron density of diamond on optical laser excitation by sum-frequency generation (Glover *et al.*, 2012 ▶). For spectroscopic experiments, an accurate knowledge of the spectral profile of the spiky FEL pulses is needed, leading to the development of new designs for single-shot spectrometers (Yabashi *et al.*, 2006 ▶; Inubushi *et al.*, 2012 ▶; Zhu *et al.*, 2012 ▶). For spectroscopy experiments at FERMI, two-colour pump–probe methods were developed (Allaria, Bencivenga *et al.*, 2013 ▶), as well as the determination of the polarization of the FEL VUV beam by molecular dichroism (Mazza *et al.*, 2014 ▶).

The high peak intensity of FELs has enabled, for the first time, studies of nonlinear effects in the VUV and soft X-ray regime, imposing new challenges for theoreticians and providing the basis for flux density limits for all other methods trying to obtain static and dynamic structural information by FELs (Wabnitz *et al.*, 2002 ▶, 2005 ▶; Sorokin *et al.*, 2007 ▶; Young *et al.*, 2010 ▶; Berrah *et al.*, 2011 ▶; Rudek *et al.*, 2012 ▶; Hishikawa *et al.*, 2011 ▶; Fukuzawa *et al.*, 2013 ▶; Tamasaku *et al.*, 2013 ▶).

At present third-generation SR sources, radiation damage is a severe issue, especially for sensitive samples like crystals of macromolecular compounds (Zeldin *et al.*, 2013 ▶) with lifetimes of less than a few seconds in a focused monochromatic beam, even at liquid-nitrogen temperatures. First calculations on the development of radiation-damage effects under the conditions of a FEL pulse indicated that one cannot avoid the primary damage caused by a direct inelastic event but that the cascade of subsequent reactions and the unavoidable Coulomb explosion takes place on timescales of several tens to hundreds of femtoseconds (Neutze *et al.*, 2000 ▶). Thus, radiation-damage effects developing on timescales longer then the FEL pulse length will be invisible in data for samples exposed to only a single pulse or for samples that can be exchanged fast enough. This scheme was first proven experimentally in CDI experiments at FLASH (Chapman *et al.*, 2006 ▶; Barty *et al.*, 2008 ▶) and later verified for crystallographic diffraction experiments, at close to atomic resolution, of micro- and nanocrystals of photosystem I at LCLS (Chapman *et al.*, 2011 ▶). This method, known under the name ‘serial femtosecond X-ray nanocrystallography’ (Chapman *et al.*, 2012 ▶; Boutet *et al.*, 2012 ▶; Kern *et al.*, 2012 ▶; Johansson *et al.*, 2013 ▶; Hirata *et al.*, 2014 ▶), bears the potential to overcome one of the main bottlenecks in the structure-solution pipeline, namely the need to grow crystals large enough for normal diffraction experiments. The crystals used in these experiments were as small as 10–20 unit cells in each dimension. Further milestones in this field were the first determination of a partly unknown structure from nanocrystals of cathepsin B grown *in vivo* (Redecke *et al.*, 2013 ▶) and of a G-protein coupled receptor grown in the lipid cubic phase (Yu *et al.*, 2013 ▶). For the *de novo* solution of protein structures at FELs, exploitation of the anomalous signal has been investigated (Barends *et al.*, 2013 ▶, 2014 ▶). A first proof-of-principle experiment on two-dimensional protein crystals has also been successfully carried out (Frank *et al.*, 2014 ▶).

First attempts on single particles of a huge virus seem to be promising (Seibert *et al.*, 2012 ▶), but for this case the competition from cryo-electron microscopy is significant and the question of which fields FELs will have an advantage in needs to be explored. This will certainly be the case for fast time-resolved studies of excited states. The potential for imaging larger objects using coherent FEL experiments becomes obvious in the imaging of living cells inside micro-liquid enclosures (Kimura *et al.*, 2014 ▶) and by the combination of CDI at a SR source and a FEL for the determination of the structure of macromolecular nanostructures (Gallagher-Jones *et al.*, 2014 ▶).

All the crystallographic experiments described above used the ultra-short FEL pulses only to minimize radiation damage. The true strength of FELs will be exploited for time-resolved studies in imaging and diffraction experiments. Early attempts at FLASH (Chapman *et al.*, 2007 ▶) established a method named ‘time-delay holography’, employing a back-reflecting multilayer for a time-delayed signal. Other experiments used a split-and-delay unit for two soft X-ray exposures with time delays down to 25 fs (Günther *et al.*, 2011 ▶). By resonant soft X-ray diffraction using the third-harmonic radiation from FLASH, it was possible to investigate the dynamics across the Verwey transition in magnetite on excitation with an optical laser at sub-picosecond timescales (Pontius *et al.*, 2011 ▶). Similar experiments were carried out to investigate the charge-order parameters in stripe-ordered La_1.75_Sr_0.25_NiO_4_ nickelate crystals (Chuang *et al.*, 2013 ▶). For serial femtosecond crystallography, laser pump X-ray probe schemes have been developed (Aquila *et al.*, 2012 ▶) and these allowed the study of the light-activated S_3_ state of photosystem II using double activation by green laser light (Kupitz *et al.*, 2014 ▶). Other recent time-resolved experiments at FELs have addressed the ultrafast lattice dynamics in nanometre-thick Si crystal layers by time-resolved Bragg coherent X-ray diffraction (Tanaka *et al.*, 2013 ▶) and the ultrafast structural dynamics in VO_2_ nanowires (Newton *et al.*, 2014 ▶), both at SACLA, and have probed the structure of water below the homogeneous ice nucleation temperature at ultra-fast timescales (Sellberg *et al.*, 2014 ▶) at LCLS.

After the success of serial femtosecond crystallography, it has been possible to employ the same ‘diffract/scatter before destruction’ technique for X-ray emission spectroscopy (Alonso-Mori *et al.*, 2012 ▶), opening the path for time-resolved X-ray spectroscopic methods. This technique has been applied to measure simultaneously, in femtosecond pump–probe fashion, the X-ray diffraction and emission signal at the Mn *K*-edge after laser excitation (Kern *et al.*, 2013 ▶). A dispersive XAS spectroscopy scheme for femtosecond time-resolved experiments within the FEL bandwidth has been successfully developed at SACLA (Katayama *et al.*, 2013 ▶; Obara *et al.*, 2014 ▶), allowing for the single-shot recording of an entire XANES spectrum.

There are scientific as well as technical reasons for trying to explore how fast magnetic order can be changed in solids. At FLASH, ultrafast magnetic demagnetization of a Co/Pt multilayer structure on timescales below 300 fs after laser excitation was discovered (Pfau *et al.*, 2012 ▶). In a follow-up experiment, fluence limits were established up to which the soft X-ray pulses do not alter the magnetic system by inelastic interactions with the electronic system of the sample within the pulse duration (Müller *et al.*, 2013 ▶). Similar timescales for demagnetization were found for ferrimagnetic GdFeCo during experiments at LCLS (Graves *et al.*, 2013 ▶) and again for Co/Pt multilayers at FERMI (von Schmising *et al.*, 2014 ▶). Also at LCLS, femtosecond single-shot imaging of the ferromagnetic order in Co/Pd multilayers could be visualized by resonant X-ray holography (Wang *et al.*, 2012 ▶) in the soft X-ray regime at the Co *L*_3_-edge, which also opens the path for spatially resolved investigations on femtosecond timescales.

It has always been the dream of researchers to follow chemical reactions in real time, the so-called molecular movie. In the mindset of crystallographers this means following a reaction from signals in reciprocal space, but this might not be the most efficient way of obtaining this information for small molecular systems. In reaction microscopes (Ullrich *et al.*, 2003 ▶), where fractions of molecules and electrons are independently detected, this information is often available much more easily. As an example, using this method enabled the dynamics of the ultrafast isomerization of acetylene cations initiated by a VUV pulse to be studied (Jiang *et al.*, 2010 ▶). For more information on the impact of FELs on molecular physics, the reader is referred to Ullrich *et al.* (2012 ▶) and references therein.

A thorough understanding of the processes at the surface of a catalyst is not only scientifically highly interesting but also bears a huge economic impact. At LCLS, the surface breaking of the CO bond on a Rh(0001) surface on optical laser excitation could be observed in real time by a combination of X-ray absorption and emission spectroscopy (Dell’Angela *et al.*, 2013 ▶).

The question that all crystallographers ask is, ‘Do we really need crystals anymore?’. In an X-ray experiment at LCLS, researchers diffracted the FEL beam from isolated and strongly aligned gas-phase molecules of 2,5-diiodobenzonitrile (Küpper *et al.*, 2014 ▶). This experiment was the first step towards diffractive imaging of distinct structures of individual isolated gas-phase molecules and proved to be suitable for studying the ultrafast dynamics of isolated molecules. These experiments were severely limited by the available repetition rate and will significantly benefit from upcoming higher-repetition-rate FELs driven by superconducting linacs. From the resolution achieved, it is obvious that crystals will still be needed for future studies at atomic resolution.

This section has tried to summarize some of the most important milestones that have been achieved during the last few years at FELs. Of course, the cited examples present a very subjective and rather incomplete selection, leaving out entire fields like, for example, experiments addressing warm dense matter or lattice dynamics. As is obvious from the science carried out so far at FELs, most of them make use of the short pulse duration and/or the high pulse intensity. So far, only a few experiments have directly exploited the transverse coherence properties of the beam, but all experiments eager for the ultimate highest photon flux density profit indirectly from the coherence properties of FELs, due to the possibility of focusing almost the entire beam into a diffraction-limited focus.

## 4. Summary and outlook   

Third-generation SR sources have reached an extremely high level of maturity during recent decades, and for many science fields the techniques at these sources provide an essential analytical backbone needed for further advances in science. Nevertheless, these sources have shortcomings. Their bunch length is inherently limited to a range of ∼40 ps, which makes studies on shorter timescales cumbersome. Also, the coherent fraction of third-generation SR sources is rather small, limiting any technique requiring coherent radiation or a high photon flux density in an as small as possible diffraction-limited focus.

This last issue will be addressed by various planned upgrades of existing sources towards a diffraction-limited storage ring (DLSR) in the X-ray regime. These sources can be considered as the next (fourth) generation of storage-ring sources. It is expected that, at almost the same total flux, the spectral brightness and coherent fraction of these sources will increase by roughly a factor of 30 to 100. Nanofocusing and coherence experiments that are limited at present by the available coherent flux will benefit directly from these increases. The construction of DLSRs based on multibend achromat magnetic lattices will certainly need careful engineering, with closer tolerances of the mechanical and magnetic design, but to the best of our knowledge all the required technologies are very mature and there are no obvious show stoppers in sight.

In principle, planned energy-recovery linac (ERL) driven sources will achieve the same or even higher performances than DLSRs in terms of spectral brightness and coherent fraction. At the same time, they will provide 1–2 ps short pulses at 1 GHz repetition rates for better temporal resolution in time-resolved experiments. Their electron bunches will be roughly an order of magnitude smaller in energy spread, enabling spectrally narrower undulator lines, which are especially advantageous for all studies requiring a highly monochromatic beam. However, so far the energy-recovery principle has only been demonstrated for beam powers two orders of magnitude below what would be needed for a modern X-ray source that is supposed to compete with DLSRs. Another challenge is the electron gun, which has to sustain a high beam current and a very small emittance simultaneously. Even though considerable progress has been made during the last decade, both issues will need further research and development efforts before a prototype for the X-ray regime will become available. For spectroscopic investigations requiring an incoming beam of extremely narrow bandwidth, Kim, Shvyd’ko & Reiche (2008 ▶) have proposed a very interesting scheme for an XFEL oscillator (X-FELO) at an ERL. Here, a crystal cavity with Bragg reflections at very high diffraction angles, or even at exact back reflection, is established such that the light of one bunch is used to seed or modulate the electron density of one of the next bunches, thus giving rise to a crystal cavity-based FEL of extremely narrow bandwidth. Similar schemes can also be implemented at high-repetition-rate linac-driven X-ray FELs.

The first generation of FELs based on the self-amplified spontaneous emission (SASE) principle has already opened up exciting new possibilities for the exploration of the structure, dynamics and function of matter by their extremely intense, highly coherent and very short light pulses. These sources will certainly not replace storage-ring based sources, but they enable a new observation window for properties not observable by other means, like time-resolved studies significantly below 100 fs resolution, or single-shot experiments in diffraction, scattering, imaging and spectroscopy. The next major improvement for FELs will certainly be seeding, either externally in the VUV regime or self-seeding for X-rays, for better control of the spectral properties of the FEL pulses and further amplification of the output radiation by tapering the FEL undulators after saturation. Methods exploiting the unique beam properties of FELs are still under development and in future we will certainly see considerably more mature experimental techniques, making them available to a larger user community.

All new source developments discussed above will provide a significantly higher degree of coherence compared with present storage-ring sources. These properties will not only allow for smaller and more intense nanofocused beams for all sorts of experiments, but also give the experimentalist a new and significantly better tool to study the structure and dynamics of disordered materials, or to establish a high-resolution microscope employing highly penetrating X-rays. In addition, FELs give access to extremely short timescales able to capture dynamics at the level of atoms, often during a single-pulse exposure, and enable the study of the properties of matter far away from equilibrium. These exciting new experimental possibilities will certainly provide the necessary tools that we need for mastering future grand challenges in health, sustainable energy, technology and a clean environment.

## Figures and Tables

**Table 1:** Comparison of a selection of storage-ring sources (operational, under construction, planned) The spectral brightness is given in the usual units [photons(mm^2^mrad^2^s0.1%BW)^1^]. (DBA: double-bend achromat; DW: damping wiggler; *x*BA: *x* bend achromat).

Source	Energy (GeV)	_*x*_ (nmrad)	Maximum *B*_*n*_ ( 10^20^)	Circumference (m)	_Dl*x*_ ()	Lattice
ESRF	6	4	3.0	844	500	DBA
APS	7	3.1	1.4	1104	390	DBA
SPring-8	8	2.7	2.5	1436	340	DBA
DIAMOND	3	2.7	1.5	561	340	DBA
PETRAIII	6	1	10	2408	126	DBA+DW
NSLS-II	3	0.5	30	792	62.8	DBA+DW
PETRAIII†	3	0.16		2408	20	DBA+DW
MAXIV	3	0.25	40	528	31	7BA
SIRIUS	3	0.28	20	518	35	5BA
ESRFII	6	0.16	100	844	20	7BA
APSII	6	0.07	200	1104	8	(58)BA
SPring-8II	6	0.10	100	1436	13	5BA

† These values given for PETRAIII (Kling Wanzenberg, 2013 ▶) were achieved in machine studies. They relate to a beam current of 5mA and this is not an intended operation mode.

**Table 2:** Envisaged parameters of some X-ray ERL projects (Nakamura, 2012 ▶; Bilderback *et al.*, 2010 ▶) The labels ‘HC’ and ‘HF’ denote the high-coherence and high-flux modes of operation, respectively. ‘CERL’ denotes the ERL project at CHESS.

Source	Energy (GeV)	Current (mA)	_*x*_ (pmrad)	*B*_*n*_ ( 10^22^)	Charge (pC)	_Dl*x*_ ()	Bunch length (ps)
KEK-HC	3	10	17	110	7.7	2	2
KEK-HF	3	100	170	110	77	20	2
CERL-HC	5	25	1334	10	19	3	1.5
CERL-HF	5	100	3152	10	77	5	2.1

**Table 3:** Characteristic parameters of some FELs, both currently operational and under construction (Hemminger, 2013 ▶; Pellegrini, 2013 ▶; Agapov *et al.*, 2014 ▶)

Source	Energy (GeV)	Wavelength ()	Pulse duration (fs)	Repetition rate (Hz)	BLs
FLASH	0.51.25	42450	20150	8000	2
FERMI	1.5	100650	100	50	2
LCLS	2.215	1.350	1100	120	1
SACLA	5.28.45	0.632.7	20	1060	1(5)
XFEL.EU	8.517.5	0.550	1100	27000	3(5)
SwissFEL	2.15.8	170	220	100	2
PAL FEL	410	16	926	60	2
LCLSII	4	2.562	2100	1M	2

## References

[bb4] Abela, R. *et al.* (2007). *The European X-ray Free-Electron Laser Technical Design Report*. http://xfel.desy.de/technical_information/tdr/tdr/.

[bb1] Abo-Bakr, M., Feikes, J., Holldack, K., Kuske, P., Peatman, W. B., Schade, U., Wüstefeld, G. & Hübers, H. W. (2003). *Phys. Rev. Lett.* **90**, 094801. 10.1103/PhysRevLett.90.094801 12689227

[bb2] Ackermann, W. *et al.* (2007). *Nat. Photon.* **1**, 336–342.

[bb3] Agapov, I., Geloni, G., Feng, G., Kocharyan, V., Saldin, E., Serkez, S. & Zagorodnov, I. (2014). *The Full Potential of the Baseline SASE Undulators of the European XFEL*. DESY Report 14-047. DESY, Hamburg, Germany.

[bb5] Allaria, E., Appio, R., Badano, L., Barletta, W. A., Bassanese, S., Biedron, S. G., Borga, A., Busetto, E., Castronovo, D. & Cinquegrana, P. (2012). *Nat. Photon.* **6**, 699–704.

[bb6] Allaria, E., Bencivenga, F. *et al.* (2013). *Nat. Commun.* **4**, 2476. 10.1038/ncomms3476 PMC3791458 24048228

[bb7] Allaria, E., Castronovo, D. *et al.* (2013). *Nat. Photon.* **7**, 913–918.

[bb8] Alonso-Mori, R. *et al.* (2012). *Proc. Natl Acad. Sci. USA*, **109**, 19103–19107.

[bb9] Altarelli, M., Kurta, R. P. & Vartanyants, I. A. (2010). *Phys. Rev. B*, **82**, 104207.

[bb10] Amann, J. *et al.* (2012). *Nat. Photon.* **6**, 693–698.

[bb11] Aquila, A. *et al.* (2012). *Opt. Express*, **20**, 2706–2716. 10.1364/OE.20.002706 PMC3413412 22330507

[bb12] Ayvazyan, V. *et al.* (2011). *Proceedings of FEL2011*, 22–26 August 2011, Shanghai, China, pp. 248–250. Red Hook, New York: Curran Associates.

[bb13] Bachelard, R., Mercère, P., Idir, M., Couprie, M. E., Labat, M., Chubar, O., Lambert, G., Zeitoun, P., Kimura, H., Ohashi, H., Higashiya, A., Yabashi, M., Nagasono, M., Hara, T. & Ishikawa, T. (2011). *Phys. Rev. Lett.* **106**, 234801. 10.1103/PhysRevLett.106.234801 21770510

[bb14] Barends, T. R., Foucar, L., Botha, S., Doak, R. B., Shoeman, R. L., Nass, K., Koglin, J. E., Williams, G. J., Boutet, S., Messerschmidt, M. & Schlichting, I. (2014). *Nature (London)*, **505**, 244–247. 10.1038/nature12773 24270807

[bb15] Barends, T. R. M. *et al.* (2013). *Acta Cryst.* D**69**, 838–842.

[bb16] Bartnik, A. *et al.* (2013). *Cornell Energy Recovery Linac*. Science Case and Project Definition Design Report. CHESS/Cornell University, Ithaca, New York, USA.

[bb17] Barty, A. *et al.* (2008). *Nat. Photon.* **2**, 415–419.

[bb18] Bernstein, F. C., Koetzle, T. F., Williams, G. J., Meyer, E. F., Brice, M. D., Rodgers, J. R., Kennard, O., Shimanouchi, T. & Tasumi, M. (1977). *J. Mol. Biol.* **112**, 535–542. 10.1016/s0022-2836(77)80200-3 875032

[bb19] Berrah, N. *et al.* (2011). *Proc. Natl Acad. Sci. USA*, **108**, 16912–16915.

[bb20] Beye, M. *et al.* (2012). *Appl. Phys. Lett.* **100**, 121108.

[bb21] Bilderback, D. H., Brock, J. D., Dale, D. S., Finkelstein, K. D., Pfeifer, M. A. & Gruner, S. M. (2010). *New J. Phys.* **12**, 035011.

[bb22] Bilderback, D., Elleaume, P. & Weckert, E. (2005). *J. Phys. B*, **38**, S773–S797.

[bb23] Bonifacio, R., Pellegrini, C. & Narducci, L. M. (1984). *Opt. Commun.* **50**, 373–378.

[bb24] Borland, M. (2013). *J. Phys. Conf. Ser.* **425**, 042016.

[bb25] Borland, M. (2014). *APS Report*, pp. 1–9. http://www.aps.anl.gov/Upgrade/Documents/multi-bend-achromat-lattice.pdf.

[bb26] Boutet, S. *et al.* (2012). *Science*, **337**, 362–364.

[bb27] Cammarata, M., Levantino, M., Schotte, F., Anfinrud, P. A., Ewald, F., Choi, J., Cupane, A., Wulff, M. & Ihee, H. (2008). *Nat. Methods*, **5**, 881–886. 10.1038/nmeth.1255 PMC3159148 18806790

[bb28] Chalupský, J. *et al.* (2009). *Opt. Express*, **17**, 208–217. 10.1364/oe.17.000208 19129890

[bb29] Chapman, H. N. *et al.* (2006). *Nat. Phys.* **2**, 839–843.

[bb30] Chapman, H. N. *et al.* (2011). *Nature (London)*, **470**, 73–77.

[bb31] Chapman, H. N. *et al.* (2012). *Nature (London)*, **469**, 73–77.

[bb32] Chapman, H. N. *et al.* (2007). *Nature (London)*, **448**, 676–679. 10.1038/nature06049 17687320

[bb33] Chavanne, J., Hahn, M., Kersevan, R., Kitegi, C., Penel, C. & Revol, F. (2008). *Proceedings of EPAC08*, 23–27 June 2008, Genoa, Italy, pp. 2243–2245. CERN, Geneva: JACoW.

[bb34] Cherezov, V., Rosenbaum, D. M., Hanson, M. A., Rasmussen, S. G. F., Thian, F. S., Kobilka, T. S., Choi, H.-J., Kuhn, P., Weis, W. I., Kobilka, B. K. & Stevens, R. C. (2007). *Science*, **318**, 1258–1265. 10.1126/science.1150577 PMC2583103 17962520

[bb35] Chuang, Y. D. *et al.* (2013). *Phys. Rev. Lett.* **110**, 127404.

[bb36] Clark, J. (2004). *The Science and Technology of Undulators and Wigglers.* Oxford University Press.

[bb37] Corlett, J. N. *et al.* (2012). *Proceedings of IPAC2012*, 20–25 May, 2012, New Orleans, Louisiana, USA, pp. 1762–1764. CERN, Geneva: JACoW.

[bb38] Deisenhofer, J., Epp, O., Miki, K., Huber, R. & Michel, H. (1985). *Nature (London)*, **318**, 618–624. 10.1038/318618a0 22439175

[bb39] Dell’Angela, M. *et al.* (2013). *Science*, **339**, 1302–1305. 10.1126/science.1231711 23493709

[bb40] Derbenev, Y. S., Kondratenko, A. M. & Saldin, E. L. (1982). *Nucl. Instrum. Methods Phys. Res.* **193**, 415–421.

[bb41] Diaz, A., Trtik, P., Guizar-Sicairos, M., Menzel, A., Thibault, P. & Bunk, O. (2012). *Phys. Rev. B*, **85**, 020104.

[bb42] Dierolf, M., Menzel, A., Thibault, P., Schneider, P., Kewish, C. M., Wepf, R., Bunk, O. & Pfeiffer, F. (2010). *Nature (London)*, **467**, 436–439. 10.1038/nature09419 20864997

[bb43] Dunham, B. M., Sinclair, C. K., Bazarov, I. V., Li, Y., Liu, X. & Smolenski, K. W. (2007). *Proceedings of PAC07*, 25–29 June, 2007, Albuquerque, New Mexico, USA, pp. 1224–1226. Piscataway: IEEE.

[bb44] Emma, P. *et al.* (2010). *Nat. Photon.* **4**, 641–647.

[bb45] Faatz, B. *et al.* (2010). *Proceedings of IPAC10*, 23–28 May 2010, Kyoto, Japan, pp. 2152–2154. CERN, Geneva: JACoW.

[bb46] Farvacque, L., Carmignani, N., Chavanne, J., Franchi, A., Le Bec, G., Liuzzo, S., Nash, B., Perron, T. & Raimondi, P. (2013). *Proceedings of IPAC2013*, 12–17 May 2013, Shanghai, China, pp. 79–81. CERN, Geneva: JACoW.

[bb47] Frank, M. *et al.* (2014). *IUCrJ*, **1**, 95–100. 10.1107/S2052252514001444 PMC4062087 25075325

[bb48] Fruehling, U., Wieland, M., Gensch, M., Gebert, T., Schuette, B., Krikunova, M., Kalms, R., Budzyn, F., Grimm, O., Rossbach, J., Ploenjes, E. & Drescher, M. (2009). *Nat. Photon.* **3**, 523–528.

[bb49] Fukuzawa, H. *et al.* (2013). *Phys. Rev. Lett.* **110**, 173005.

[bb50] Gahl, C., Azima, A., Beye, M., Deppe, M., Doebrich, K., Hasslinger, U., Hennies, F., Melnikov, A., Nagasono, M., Pietzsch, A., Wolf, M., Wurth, W. & Foehlisch, A. (2008). *Nat. Photon.* **2**, 165–169.

[bb51] Gallagher-Jones, M. *et al.* (2014). *Nat. Commun.* **5**, 3798. 10.1038/ncomms4798 24786694

[bb52] Ganter, R. (2010). *SwissFEL Conceptual Design Report*. Paul Scherrer Institute (PSI), Villigen, Switzerland.

[bb53] Garman, E. F. (2014). *Science*, **343**, 1102–1108. 10.1126/science.1247829 24604194

[bb54] Gati, C., Bourenkov, G., Klinge, M., Rehders, D., Stellato, F., Oberthür, D., Yefanov, O., Sommer, B. P., Mogk, S., Duszenko, M., Betzel, C., Schneider, T. R., Chapman, H. N. & Redecke, L. (2014). *IUCrJ*, **1**, 87–94. 10.1107/S2052252513033939 PMC4062088 25075324

[bb55] Gaudin, J. *et al.* (2012). *Phys. Rev. B*, **86**, 024103.

[bb56] Geloni, G., Kocharyan, V. & Saldin, E. (2010). *Self-Seeded Operation of the LCLS Hard X-ray FEL in the Long-Bunch Mode*. DESY Report 10-239. DESY, Hamburg, Germany.

[bb57] Glover, T. E., Fritz, D. M., Cammarata, M., Allison, T. K., Coh, S., Feldkamp, J. M., Lemke, H., Zhu, D., Feng, Y., Coffee, R. N., Fuchs, M., Ghimire, S., Chen, J., Shwartz, S., Reis, D. A., Harris, S. E. & Hastings, J. B. (2012). *Nature (London)*, **488**, 603–608. 10.1038/nature11340 22932384

[bb58] Graves, C. E. *et al.* (2013). *Nat. Mater.* **12**, 293–298. 10.1038/nmat3597 23503010

[bb59] Grguras, I., Maier, A. R., Behrens, C., Mazza, T., Kelly, T. J., Radcliffe, P., Dusterer, S., Kazansky, A. K., Kabachnik, N. M. & Tschentscher, T. (2012). *Nat. Photon.* **6**, 852–857.

[bb60] Grübel, G., Madsen, A. & Robert, A. (2008). *Soft Matter Characterisation*, edited by R. Borsali and R. Pecora, p. 953. Heidelberg: Springer.

[bb61] Grübel, G. & Zontone, F. (2004). *J. Alloys Compd.* **362**, 3–11.

[bb62] Gruner, S. M. & Tigner, M. (2001). Editors. *Study for a Proposed Phase I Energy Recovery Linac (ERL) Synchrotron Light Source at Cornell University*, CHESS Technical Memo 01-003 and JLAB-ACT-01-04. CHESS, Ithaca, NY, USA.

[bb63] Günther, C. M., Pfau, B., Mitzner, R., Siemer, B., Roling, S., Zacharias, H., Kutz, O., Rudolph, I., Schondelmaier, D. & Treusch, R. (2011). *Nat. Photon.* **5**, 99–102.

[bb64] Gutt, C. *et al.* (2012). *Phys. Rev. Lett.* **108**, 024801. 10.1103/PhysRevLett.108.024801 22324689

[bb65] Hama, H., Takano, S. & Isoyama, G. (1993). *Nucl. Instrum. Methods Phys. Res. A*, **329**, 29–36.

[bb66] Hanbury-Brown, R. & Twiss, R. Q. (1956). *Nature (London)*, **177**, 27–29.

[bb67] Hara, T., Tanaka, T., Kitamura, H., Bizen, T., Marechal, X., Seike, T., Kohda, T. & Matsuura, Y. (2004). *Phys. Rev. ST Accel. Beams*, **7**, 050702.

[bb68] Hara, T., Tanaka, T., Tanabe, T., Maréchal, X.-M., Kitamura, H., Elleaume, P., Morrison, B., Chavanne, J., Van Vaerenbergh, P. & Schmidt, D. (1998). *J. Synchrotron Rad.* **5**, 406–408. 10.1107/S0909049597014325 15263526

[bb69] Harmand, M., Coffee, R., Bionta, M. R., Chollet, M., French, D., Zhu, D., Fritz, D. M., Lemke, H. T., Medvedev, N. & Ziaja, B. (2013). *Nat. Photon.* **7**, 215–218.

[bb70] Hau-Riege, S. P. *et al.* (2007). *Appl. Phys. Lett.* **90**, 173128.

[bb71] Hemminger, J. (2013). *Future Light Sources Report*. BESAC approved. Washington DC: BESAC-DOE.

[bb72] Hirata, K. *et al.* (2014). *Nat. Methods*, **11**, 734–736. 10.1038/nmeth.2962 24813624

[bb73] Hishikawa, A., Fushitani, M., Hikosaka, Y., Matsuda, A., Liu, C. N., Morishita, T., Shigemasa, E., Nagasono, M., Tono, K., Togashi, T., Ohashi, H., Kimura, H., Senba, Y., Yabashi, M. & Ishikawa, T. (2011). *Phys. Rev. Lett.* **107**, 243003. 10.1103/PhysRevLett.107.243003 22242995

[bb74] Hoffstaetter, G. H. *et al.* (2013). *Proceedings of IPAC2013*, 12–17 May 2013, Shanghai, China, pp. 2447–2449. CERN, Geneva: JACoW.

[bb75] Holler, M., Diaz, A., Guizar-Sicairos, M., Karvinen, P., Färm, E., Härkönen, E., Ritala, M., Menzel, A., Raabe, J. & Bunk, O. (2014). *Sci. Rep.* **4**, 3857. 10.1038/srep03857 PMC3900995 24457289

[bb76] Inubushi, Y., Tono, K., Togashi, T., Sato, T., Hatsui, T., Kameshima, T., Togawa, K., Hara, T., Tanaka, T., Tanaka, H., Ishikawa, T. & Yabashi, M. (2012). *Phys. Rev. Lett.* **109**, 144801. 10.1103/PhysRevLett.109.144801 23083249

[bb77] Ishikawa, T. *et al.* (2012). *Nat. Photon.* **6**, 540–544.

[bb78] Ivanyushenkov, Y. & Harkay, K. (2014). *Proceedings of IPAC2014*, 15–20 June, 2014, Dresden, Germany, p. 2053. CERN, Geneva: JACoW.

[bb79] Jiang, Y. H., Rudenko, A., Herrwerth, O., Foucar, L., Kurka, M., Kühnel, K. U., Lezius, M., Kling, M. F., van Tilborg, J., Belkacem, A., Ueda, K., Düsterer, S., Treusch, R., Schröter, C. D., Moshammer, R. & Ullrich, J. (2010). *Phys. Rev. Lett.* **105**, 263002. 10.1103/PhysRevLett.105.263002 21231652

[bb80] Johansson, L. C. *et al.* (2013). *Nat. Commun.* **4**, 2911. 10.1038/ncomms3911 PMC3905732 24352554

[bb81] Katayama, T., Inubushi, Y., Obara, Y., Sato, T., Togashi, T., Tono, K., Hatsui, T., Kameshima, T., Bhattacharya, A., Ogi, Y., Kurahashi, N., Misawa, K., Suzuki, T. & Yabashi, M. (2013). *Appl. Phys. Lett.* **103**, 131105.

[bb82] Kern, J. *et al.* (2012). *Proc. Natl Acad. Sci. USA*, **109**, 9721–9726.

[bb83] Kern, J. *et al.* (2013). *Science*, **340**, 491–495.

[bb84] Khorsand, A. R. *et al.* (2010). *Opt. Express*, **18**, 700–712. 10.1364/OE.18.000700 20173890

[bb86] Kim, K.-J., Shvyd’ko, Y. & Reiche, S. (2008). *Phys. Rev. Lett.* **100**, 244802. 10.1103/PhysRevLett.100.244802 18643591

[bb85] Kim, M. G., Choi, J., Lee, T. Y., Kang, H. S., Yim, C.-M. & Ko, I. S. (2008). *J. Korean Phys. Soc.* **53**, 3741–3743.

[bb87] Kimura, T., Joti, Y., Shibuya, A., Song, C., Kim, S., Tono, K., Yabashi, M., Tamakoshi, M., Moriya, T., Oshima, T., Ishikawa, T., Bessho, Y. & Nishino, Y. (2014). *Nat. Commun.* **5**, 3052. 10.1038/ncomms4052 PMC3896756 24394916

[bb88] Kling, A. & Wanzenberg, R. (2013). *Beam Dynam. Newsl.* **62**, 235–243.

[bb89] Kondratenko, A. M. & Saldin, E. L. (1980). *Part. Accel.* **10**, 207–216.

[bb90] Korff Schmising, C. von, Pfau, B., Schneider, M., Günther, C. M., Giovannella, M., Perron, J., Vodungbo, B., Müller, L., Capotondi, F., Pedersoli, E., Mahne, N., Luning, J. & Eisebitt, S. (2014). *Phys. Rev. Lett.* **112**, 217203.

[bb91] Koyama, T., Yumoto, H., Senba, Y., Tono, K., Sato, T., Togashi, T., Inubushi, Y., Katayama, T., Kim, J., Matsuyama, S., Mimura, H., Yabashi, M., Yamauchi, K., Ohashi, H. & Ishikawa, T. (2013). *Opt. Express*, **21**, 15382–15388. 10.1364/OE.21.015382 23842324

[bb92] Kupitz, C. *et al.* (2014). *Nature (London)*, **513**, 261–265.

[bb93] Küpper, J. *et al.* (2014). *Phys. Rev. Lett.* **112**, 083002.

[bb94] Kurta, R. P., Altarelli, M., Weckert, E. & Vartanyants, I. A. (2012). *Phys. Rev. B*, **85**, 184204.

[bb95] Kurta, R. P., Dronyak, R., Altarelli, M., Weckert, E. & Vartanyants, I. A. (2013). *New J. Phys.* **15**, 013059.

[bb96] Kurta, R. P., Ostrovskii, B. I., Singer, A., Gorobtsov, O. Y., Shabalin, A., Dzhigaev, D., Yefanov, O. M., Zozulya, A. V., Sprung, M. & Vartanyants, I. A. (2013). *Phys. Rev. E*, **88**, 044501. 10.1103/PhysRevE.88.044501 24229307

[bb97] Lehmkühler, F., Gutt, C., Fischer, B., Schroer, M. A., Sikorski, M., Song, S., Roseker, W., Glownia, J., Chollet, M., Nelson, S., Tono, K., Katayama, T., Yabashi, M., Ishikawa, T., Robert, A. & Grübel, G. (2014). *Sci. Rep.* **4**, 5234. 10.1038/srep05234 PMC4050387 24913261

[bb98] LNLS (2014). *SIRIUS: The New Brazilian Synchrotron Light Source*. Brazilian Synchrotron Light Laboratory, Campinas, SP, Brazil. http://lnls.cnpem.br/sirius/

[bb99] MAX IV (2014). *Detailed Design Report on the MAX IV Facility*, https://www.maxlab.lu.se/maxlab/max4/index.html.

[bb100] Mayes, C. & Hoffstaetter, G. (2010). *Proceedings of IPAC10*, 23–28 May 2010, Kyoto, Japan, pp. 2353–2355. CERN, Geneva: JACoW.

[bb101] Mazza, T. *et al.* (2014). *Nat. Commun.* **5**, 3648. 10.1038/ncomms4648 24736496

[bb102] Merminga, L. (2007). *Proceedings of PAC07*, 25–29 June, 2007, Albuquerque, New Mexico, USA, pp. 22–26. Piscataway: IEEE.

[bb103] Miao, J., Kirz, J. & Sayre, D. (2000). *Acta Cryst.* D**56**, 1312–1315. 10.1107/s0907444900008970 10998627

[bb104] Mimura, H., Morita, S., Kimura, T., Yamakawa, D., Lin, W., Uehara, Y., Matsuyama, S., Yumoto, H., Ohashi, H., Tamasaku, K., Nishino, Y., Yabashi, M., Ishikawa, T., Ohmori, H. & Yamauchi, K. (2008). *Rev. Sci. Instrum.* **79**, 083104. 10.1063/1.2964928 19044333

[bb105] Mimura, H., Yumoto, H., Matsuyama, S., Koyama, T., Tono, K., Inubushi, Y., Togashi, T., Sato, T., Kim, J., Fukui, R., Sano, Y., Yabashi, M., Ohashi, H., Ishikawa, T. & Yamauchi, K. (2014). *Nat. Commun.* **5**, 3539. 10.1038/ncomms4539 24781443

[bb106] Motz, H. (1951). *J. Appl. Phys.* **22**, 527–535.

[bb107] Müller, L. *et al.* (2013). *Phys. Rev. Lett.* **110**, 234801.

[bb108] Murphy, J. B. & Pellegrini, C. (1985). *Nucl. Instrum. Methods A*, **237**, 159–167.

[bb109] Nakamura, N. (2012). *Proceedings of IPAC2012*, 20–25 May, 2012, New Orleans, Louisiana, USA, pp. 1040–1044. CERN, Geneva: JACoW.

[bb110] Neutze, R., Wouts, R., van der Spoel, D., Weckert, E. & Hajdu, J. (2000). *Nature (London)*, **406**, 752–757. 10.1038/35021099 10963603

[bb111] Newton, M. C., Sao, M., Fujisawa, Y., Onitsuka, R., Kawaguchi, T., Tokuda, K., Sato, T., Togashi, T., Yabashi, M., Ishikawa, T., Ichitsubo, T., Matsubara, E., Tanaka, Y. & Nishino, Y. (2014). *Nano Lett.* **14**, 2413–2418. 10.1021/nl500072d 24742218

[bb112] NSLS-II (2010). *NSLS-II Source Properties and Floor Layout*. NSLS-II, Brookhaven National Laboratory, New York, USA. http://www.bnl.gov/ps/docs/pdf/SourceProperties.pdf

[bb113] Obara, Y., Katayama, T., Ogi, Y., Suzuki, T., Kurahashi, N., Karashima, S., Chiba, Y., Isokawa, Y., Togashi, T., Inubushi, Y., Yabashi, M., Suzuki, T. & Misawa, K. (2014). *Opt. Express*, **22**, 1105–1113. 10.1364/OE.22.001105 24515070

[bb114] Orzechowski, T. J., Anderson, B. R., Clark, J. C., Fawley, W. M., Paul, A. C., Prosnitz, D., Scharlemann, E. T., Yarema, S. M., Hopkins, D. B., Sessler, A. M. & Wurtele, J. S. (1986). *Phys. Rev. Lett.* **57**, 2172–2175. 10.1103/PhysRevLett.57.2172 10033654

[bb115] Pellegrini, C. (2013). *Proceedings of NAPAC13*, 29 September–4 October 2013, Pasadena, California, USA, pp. 1–34. CERN, Geneva: JACoW.

[bb116] Pfau, B. *et al.* (2012). *Nat. Commun.* **3**, 1100. 10.1038/ncomms2108 PMC3493637 23033076

[bb117] Pontius, N. *et al.* (2011). *Appl. Phys. Lett.* **98**, 182504.

[bb118] Redecke, L. *et al.* (2013). *Science*, **339**, 227–230.

[bb119] Reiche, S. (2013). *Proceedings of IPAC2013*, 12–17 May 2013, Shanghai, China, pp. 2063–2067. CERN, Geneva: JACoW.

[bb120] Revol, J. L. *et al.* (2013). *Proceedings of IPAC2013*, 12–17 May 2013, Shanghai, China, pp. 1140–1142. CERN, Geneva: JACoW.

[bb121] Riedel, R., Al-Shemmary, A., Gensch, M., Golz, T., Harmand, M., Medvedev, N., Prandolini, M. J., Sokolowski-Tinten, K., Toleikis, S., Wegner, U., Ziaja, B., Stojanovic, N. & Tavella, F. (2013). *Nat. Commun.* **4**, 1731. 10.1038/ncomms2754 23591898

[bb122] Robin, D., Forest, E., Pellegrini, C. & Amiry, A. (1993). *Phys. Rev. E*, **48**, 2149–2156. 10.1103/physreve.48.2149 9960832

[bb123] Rodenburg, J. M., Hurst, A. C., Cullis, A. G., Dobson, B. R., Pfeiffer, F., Bunk, O., David, C., Jefimovs, K. & Johnson, I. (2007). *Phys. Rev. Lett.* **98**, 034801. 10.1103/PhysRevLett.98.034801 17358687

[bb124] Rossmanith, R., Moser, H. O., Geisler, A., Hobl, A., Krischel, D. & Schillo, M. (2002). *Proceedings of EPAC 2002*, 3–7 June 2002, Paris, France, pp. 2628–2630. CERN, Geneva: JACoW.

[bb125] Rudek, B. *et al.* (2012). *Nat. Photon.* **6**, 858–865.

[bb126] Rutishauser, S., Samoylova, L., Krzywinski, J., Bunk, O., Grünert, J., Sinn, H., Cammarata, M., Fritz, D. M. & David, C. (2012). *Nat. Commun.* **3**, 947. 10.1038/ncomms1950 22781760

[bb127] Saldin, E. L., Schneidmiller, E. A. & Yurkov, M. V. (1998*a*). *Opt. Commun.* **148**, 383–403.

[bb128] Saldin, E. L., Schneidmiller, E. A. & Yurkov, M. V. (1998*b*). *Nucl. Inst. Methods Phys. Res. A*, **407**, 291–295.

[bb129] Saldin, E. L., Schneidmiller, E. A. & Yurkov, M. V. (2008*a*). *Opt. Commun.* **281**, 1179–1188.

[bb130] Saldin, E. L., Schneidmiller, E. A. & Yurkov, M. V. (2008*b*). *Opt. Commun.* **281**, 4727–4734.

[bb131] Schluenzen, F., Tocilj, A., Zarivach, R., Harms, J., Gluehmann, M., Janell, D., Bashan, A., Bartels, H., Agmon, I., Franceschi, F. & Yonath, A. (2000). *Cell*, **102**, 615–623. 10.1016/s0092-8674(00)00084-2 11007480

[bb132] Schmüser, P., Dohlus, M., Rossbach, J. & Behrens, C. (2014). *Free Electron Lasers in the Ultraviolet and X-ray Regime: Physical Principles, Experimental Results, Technical Realization*, 2nd ed. Berlin: Springer-Verlag.

[bb133] Schreiber, S. (2010). *Rev. Accel. Sci. Technol.* **3**, 93–120.

[bb134] Schroer, C. G., Boye, P., Feldkamp, J. M., Patommel, J., Schropp, A., Schwab, A., Stephan, S., Burghammer, M., Schöder, S. & Riekel, C. (2008). *Phys. Rev. Lett.* **101**, 090801. 10.1103/PhysRevLett.101.090801 18851597

[bb135] Schroer, C. G. & Falkenberg, G. (2014). *J. Synchrotron Rad.* **21**, 996–1005. 10.1107/S1600577514016269 PMC4151680 25177988

[bb136] Schropp, A., Hoppe, R., Meier, V., Patommel, J., Seiboth, F., Lee, H. J., Nagler, B., Galtier, E. C., Arnold, B., Zastrau, U., Hastings, J. B., Nilsson, D., Uhlén, F., Vogt, U., Hertz, H. M. & Schroer, C. G. (2013). *Sci. Rep.* **3**, 1633. 10.1038/srep01633 PMC3620670 23567281

[bb137] Schropp, A., Hoppe, R., Patommel, J., Samberg, D., Seiboth, F., Stephan, S., Wellenreuther, G., Falkenberg, G. & Schroer, C. G. (2012). *Appl. Phys. Lett.* **100**, 253112.

[bb138] Seibert, M. M. *et al.* (2012). *Nature (London)*, **469**, 78–81.

[bb139] Sellberg, J. A. *et al.* (2014). *Nature (London)*, **510**, 381–384.

[bb140] Serkez, S., Kocharyan, V., Saldin, E., Zagorodnov, I. & Geloni, G. (2013). *Proposal to Generate 10 TW Level Femtosecond X-ray Pulses from a Baseline Undulator in Conventional SASE Regime at the European XFEL*. DESY Report 13-138. DESY, Hamburg, Germany.

[bb141] Siggins, T., Sinclair, C., Bohn, C., Bullard, D., Douglas, D., Grippo, A., Gubeli, J., Krafft, G. A. & Yunn, B. (2001). *Nucl. Inst. Methods Phys. Res. A*, **475**, 549–553.

[bb142] Singer, A., Lorenz, U., Sorgenfrei, F., Gerasimova, N., Gulden, J., Yefanov, O. M., Kurta, R. P., Shabalin, A., Dronyak, R., Treusch, R., Kocharyan, V., Weckert, E., Wurth, W. & Vartanyants, I. A. (2013). *Phys. Rev. Lett.* **111**, 034802. 10.1103/PhysRevLett.111.034802 23909331

[bb143] Singer, A. *et al.* (2012). *Opt. Express*, **20**, 17480–17495. 10.1364/OE.20.017480 23038301

[bb144] Singer, A. & Vartanyants, I. A. (2014). *J. Synchrotron Rad.* **21**, 5–15. 10.1107/S1600577513023850 PMC3874016 24365911

[bb145] Singer, A., Vartanyants, I. A., Kuhlmann, M., Duesterer, S., Treusch, R. & Feldhaus, J. (2008). *Phys. Rev. Lett.* **101**, 254801. 10.1103/PhysRevLett.101.254801 19113716

[bb146] Sobierajski, R. *et al.* (2011). *Opt. Express*, **19**, 193–205. 10.1364/OE.19.000193 21263557

[bb147] Sorokin, A. A., Bobashev, S. V., Feigl, T., Tiedtke, K., Wabnitz, H. & Richter, M. (2007). *Phys. Rev. Lett.* **99**, 213002. 10.1103/PhysRevLett.99.213002 18233213

[bb148] Steeg, B., Juha, L., Feldhaus, J., Jacobi, S., Sobierajski, R., Michaelsen, C., Andrejczuk, A. & Krzywinski, J. (2004). *Appl. Phys. Lett.* **84**, 657.

[bb149] Stojanovic, N. *et al.* (2006). *Appl. Phys. Lett.* **89**, 241909.

[bb150] Tamasaku, K., Nagasono, M., Iwayama, H., Shigemasa, E., Inubushi, Y., Tanaka, T., Tono, K., Togashi, T., Sato, T., Katayama, T., Kameshima, T., Hatsui, T., Yabashi, M. & Ishikawa, T. (2013). *Phys. Rev. Lett.* **111**, 043001. 10.1103/PhysRevLett.111.043001 23931361

[bb151] Tanaka, Y., Ito, K., Nakatani, T., Onitsuka, R., Newton, M., Sato, T., Togashi, T., Yabashi, M., Kawaguchi, T., Shimada, K., Tokuda, K., Takahashi, I., Ichitsubo, T., Matsubara, E. & Nishino, Y. (2013). *J. Ceram. Soc. Jpn*, **121**, 283–286.

[bb152] Tavella, F., Stojanovic, N., Geloni, G. & Gensch, M. (2011). *Nat. Photon.* **5**, 162–165.

[bb153] Thompson, A., Attwood, D., Gullikson, E., Howells, M., Kim, K.-J., Kirz, J., Kortright, J., Lindau, I., Liu, Y., Pianetta, P., Robinson, A., Scofield, J., James, U., Williams, G. & Winick, H. (2009). *X-ray Data Booklet*, 3rd ed. Berkeley: Lawrence Berkeley National Laboratory.

[bb154] Tigner, M. (1965). *Nuovo Cimento*, **37**, 1228–1231.

[bb155] Trtik, P., Diaz, A., Guizar-Sicairos, M., Menzel, A. & Bunk, O. (2013). *Cem. Concr. Compos.* **36**, 71–77.

[bb156] Uhlén, F., Nilsson, D., Holmberg, A., Hertz, H. M., Schroer, C. G., Seiboth, F., Patommel, J., Meier, V., Hoppe, R., Schropp, A., Lee, H. J., Nagler, B., Galtier, E., Krzywinski, J., Sinn, H. & Vogt, U. (2013). *Opt. Express*, **21**, 8051–8061. 10.1364/OE.21.008051 23571895

[bb157] Ullrich, J., Moshammer, R., Dorn, A., Dorner, R., Schmidt, L. & Schniidt-Bocking, H. (2003). *Rep. Prog. Phys.* **66**, 1463–1545.

[bb158] Ullrich, J., Rudenko, A. & Moshammer, R. (2012). *Annu. Rev. Phys. Chem.* **63**, 635–660. 10.1146/annurev-physchem-032511-143720 22404584

[bb159] Vartanyants, I. A. & Singer, A. (2010). *New J. Phys.* **12**, 035004.

[bb160] Vartanyants, I. A. *et al.* (2011). *Phys. Rev. Lett.* **107**, 144801.

[bb161] Vashchenko, G. (2013). PhD thesis, University of Hamburg, Germany.

[bb162] Veen, F. van der & Pfeiffer, F. (2004). *J. Phys.* **16**, 5003–5030.

[bb163] Wabnitz, H. *et al.* (2002). *Nature (London)*, **420**, 482–485.

[bb164] Wabnitz, H., de Castro, A. R., Gürtler, P., Laarmann, T., Laasch, W., Schulz, J. & Möller, T. (2005). *Phys. Rev. Lett.* **94**, 023001. 10.1103/PhysRevLett.94.023001 15698168

[bb165] Wang, T. *et al.* (2012). *Phys. Rev. Lett.* **108**, 267403. 10.1103/PhysRevLett.108.267403 23005013

[bb166] Weckert, E., Balewski, K., Brefeld, W., Decking, W., Drube, W., Franz, H., Gürtler, P., Hahn, U., Pflüger, J., Schulte-Schrepping, H., Tischer, M. & Schneider, J. (2004). *AIP Conf. Proc.* **705**, 73–76.

[bb167] Wiedemann, H. (1988). *Nucl. Instrum. Methods Phys. Res. A*, **266**, 24–31.

[bb168] Wiedemann, H. (2007). *Particle Accelerator Physics.* Heidelberg: Springer Science and Business Media.

[bb169] Williams, G. J., Pfeifer, M. A., Vartanyants, I. A. & Robinson, I. K. (2003). *Phys. Rev. Lett.* **90**, 175501. 10.1103/PhysRevLett.90.175501 12786079

[bb170] Wochner, P., Gutt, C., Autenrieth, T., Demmer, T., Bugaev, V., Ortiz, A. D., Duri, A., Zontone, F., Grübel, G. & Dosch, H. (2009). *Proc. Natl Acad. Sci. USA*, **106**, 11511–11514. 10.1073/pnas.0905337106 PMC2703671 20716512

[bb171] Wulff, M., Kong, Q., Cammarata, M., Lo Russo, M., Anfinrud, P., Schotte, F., Lorenc, M., Ihee, H., Kim, T. K. & Plech, A. (2007). *AIP Conf. Proc.* **879**, 1187–1194.

[bb172] Yabashi, M., Hastings, J. B., Zolotorev, M. S., Mimura, H., Yumoto, H., Matsuyama, S., Yamauchi, K. & Ishikawa, T. (2006). *Phys. Rev. Lett.* **97**, 084802. 10.1103/PhysRevLett.97.084802 17026309

[bb173] Young, L. *et al.* (2010). *Nature (London)*, **466**, 56–61.

[bb174] Yu, L. H. (1991). *Phys. Rev. A*, **44**, 5178–5193. 10.1103/physreva.44.5178 9906572

[bb175] Yu, C., Zhang, Y.-L., Pan, W.-W., Li, X.-M., Wang, Z.-W., Ge, Z.-J., Zhou, J.-J., Cang, Y., Tong, C. & Sun, Q.-Y. (2013). *Science*, **342**, 1518–1521. 10.1126/science.1244587 24357321

[bb176] Yumoto, H. *et al.* (2012). *Nat. Photon.* **7**, 43–47.

[bb177] Zeldin, O. B., Brockhauser, S., Bremridge, J., Holton, J. M. & Garman, E. F. (2013). *Proc. Natl Acad. Sci. USA*, **110**, 20551–20556. 10.1073/pnas.1315879110 PMC3870734 24297937

[bb178] Zholents, A. A. & Zolotorev, M. S. (1996). *Phys. Rev. Lett.* **76**, 912–915. 10.1103/PhysRevLett.76.912 10061583

[bb179] Zhu, D., Cammarata, M., Feldkamp, J. M., Fritz, D. M., Hastings, J. B., Lee, S., Lemke, H. T., Robert, A., Turner, J. L. & Feng, Y. (2012). *Appl. Phys. Lett.* **101**, 034103.

